# HLA-DRB1 haplotypes predict cardiovascular mortality in inflammatory polyarthritis independent of CRP and anti-CCP status

**DOI:** 10.1186/s13075-022-02775-0

**Published:** 2022-04-25

**Authors:** Seema Sharma, Darren Plant, John Bowes, Alex Macgregor, Suzanne Verstappen, Anne Barton, Sebastien Viatte

**Affiliations:** 1 Centre for Genetics and Genomics Versus Arthritis, Centre for Musculoskeletal Research, Manchester Academic Health Science Centre, The University of Manchester, Manchester, Oxford Road, Manchester, M13 9PT UK; 2 Rheumatology Department, Norfolk and Norwich University Hospitals NHS Trust, Norwich, NR4 7UY UK; 3 Norwich Medical School, University of East Anglia Faculty of Medicine and Health Sciences, Norwich, NR4 7TJ UK; 4 NIHR Manchester Musculoskeletal Biomedical Research Unit, Central Manchester NHS Foundation Trust, Manchester Academic Health Sciences Centre, Grafton Street, Manchester, M13 9WL UK; 5 Lydia Becker Institute of Immunology and Inflammation, Faculty of Biology, Medicine and Health, The University of Manchester, Manchester, M13 9PL UK

**Keywords:** Cardiovascular mortality, Genetic biomarkers, HLA-DRB1, Rheumatoid arthritis, Anti-citrullinated protein antibodies

## Abstract

**Background:**

Haplotypes defined by amino acids at HLA-DRB1 positions 11, 71 and 74 associated with susceptibility to rheumatoid arthritis (RA) are associated with radiological outcome, anti-TNF response and all cause-mortality in RA. RA is associated with cardiovascular (CV) morbidity and mortality, but the increased prevalence of risk factors of CV disease in RA only partially explains this association. The aim of this study was to investigate whether amino acids at positions 11, 71 and 74 of HLA-DRB1 are associated with cardiovascular (CV) mortality in inflammatory polyarthritis (IP).

**Methods:**

The Norfolk Arthritis Register (NOAR) is an incidence register of IP: recruitment 1990–2007, final follow-up 2011. Two thousand five hundred fourteen patients had available genetic and mortality data. Amino acids at positions 11, 71 and 74 of HLA-DRB1 were determined. Univariate Cox proportional hazard models were applied to assess the association of genetic markers and both all-cause mortality and cardiovascular mortality.

**Results:**

Among 2514 participants, 643 (25.6%) died during the study, and 343 (53.3%) of these deaths were attributed to CV causes. One thousand six hundred fifty (65.6%) participants were female, 709 (32.3%) were anti-CCP-positive and the median age of participants was 54.

HLA-DRB1 haplotypes associated with susceptibility to rheumatoid arthritis (RA) consistently show the same magnitude and direction of association for overall and CV mortality in IP. For example, the SEA-haplotype, associated with the lowest susceptibility to RA, and the best radiographic outcome, was found to be associated with decreased CV mortality (HR 0.67, 95% CI 0.47, 0.91, *p*=0.023). Mediation analysis revealed associations were independent of anti-CCP status.

**Conclusions:**

HLA-DRB1 haplotypes associated with susceptibility to RA also predispose to increased risk of CV mortality in IP, independent of known CV risk factors. Associations were independent of anti-CCP status, which suggests in the future, genetic factors will add to the prediction of risk of cardiovascular mortality beyond serological markers.

## Key messages

- Positions 11, 71 and 74 of HLA-DRB1 are associated with cardiovascular mortality in inflammatory polyarthritis.
- Position 11, which has not been previously implicated with cardiovascular mortality, shows the strongest association. This effect is independent of anti-CCP status and CRP at baseline.
- The findings suggest that genetic factors may to the prediction of risk of cardiovascular mortality beyond traditional cardiovascular risk factors and serological markers.

## Introduction

Rheumatoid arthritis (RA) is associated with cardiovascular (CV) morbidity and mortality [1]. Identifying those at greatest risk could have the potential to enable better risk stratification at disease onset and target interventions to those of greatest need. Traditional CV risk factors amongst patients with RA do not completely explain the risk of premature death noted in this patient group [2]. It is thought that increased inflammation may promote atherosclerosis, a notion supported by the finding that those with greater disease burden are most at risk of all-cause mortality [3, 4]. Genetic factors have also been implicated in the mechanism underlying the prevalence of CV mortality in RA [5, 6].

RA genetic susceptibility alleles at the HLA-DRB1 gene locus encoding for a conserved amino acid sequence at positions 70–74 are known as “the shared epitope” (SE). The SE alleles, have been found in previous studies to be associated with mortality in RA, a substantial component of which was through CV disease [3, 7].

Recently, amino acids at HLA-DRB1 position 11, outside the SE, were shown to be the strongest genetic predictors of RA susceptibility [8–11]. Additionally, we could show that 16 haplotypes, as defined by amino acid positions 11, 71 and 74 of HLA-DRB1, were associated with radiological outcome, anti-TNF response and all cause-mortality in RA and that these haplotypes can be ranked in a hierarchy according to risk [12]. We hypothesise that these haplotypes are also associated with CV mortality.

The aim of this study was to identify whether this genetic information could add to clinical predictors at baseline, to predict those at the highest risk of cardiovascular mortality.

## Patients and methods

### Cohort

The Norfolk Arthritis Register (NOAR) is a primary care-based inception cohort of patients with IP defined as a minimum of 2 swollen joints for a period of at least 4 weeks [13]. Patients were recruited in 1989 and followed up for up to 20 years. NOAR patients with at least 2 years of follow-up time with available mortality and genetic data were included in this study [6]. Ethical approval was granted by the Norwich Research Ethics committee. All patients were recruited following informed consent.

### Genotyping

A semiautomated, reverse dot-blot method was used in HLA typing [14]. All samples of sufficient quality were additionally genotyped using a single-nucleotide polymorphism microarray (Illumina Infinium Immunochip) and imputed at the amino acid resolution [15]. Amino acids at positions 11, 71 and 74 were determined, combinations of which result in a total of sixteen possible haplotypes. These haplotypes have been previously grouped into four groups as defined previously [12]. Full-genotyping methodology, including nomenclature and a list of possible amino acids at DRB1 positions 11, 71, or 74, can be found elsewhere [12].

### Serology

Anticyclic citrullinated peptide antibody positivity (anti-CCP2) was determined with the CCP2 assay; Axis-Shield DIASTA kit (Axis-Shield Dundee, UK), where > 5 U/ml was defined as positive.

### Mortality

Data on all-cause mortality and CV mortality was provided by the Office for National Statistics, as described previously, where CV mortality was recognised as CVD mentioned on the death certification, using the International Classification of Diseases Tenth Revision [7].

### Statistical analysis

Univariate Cox proportional hazard models were applied to assess the association of genetic markers and both all-cause mortality and CV mortality. Models for all-cause and CV mortality were adjusted for available CV risk factors: obesity, gender and evidence of hypertension (defined by self-reported co-prescription of antihypertensive agents). These covariates were identified using forward stepwise regression. Variables found to be collinear were not included in the final analysis. These were smoking status, evidence of diabetes (defined as self-reported co-prescription of diabetes medications), age at baseline assessment and statin use. Data on all variables was collected at baseline assessment.

Hazard models were firstly applied to the entire cohort of patients with IP and then restricted to patients who fulfilled the 1987 classification criteria for RA. When calculating differences between highest and lowest risk genetic factors, bivariate analysis was used. Results are reported as hazard ratios with 95% confidence intervals.

Lastly, a multivariate cox-proportional hazard model was applied to the 3 most frequent haplotypes occurring in the NOAR cohort, defined by an allele frequency of over 12% to test their association with CV mortality. A one-tailed *p* value was calculated using a linear regression model to determine the association between effect sizes *(β coefficients)* of susceptibility and CV mortality. All analysis was performed using STATA/IC 14.0.

### Mediation analysis

Mediation analysis was performed to determine whether the genetic effects of CV mortality were due to intermediate parameters (anti-CCP, CRP) [16]. This analysis was performed as per principles according to Baron and Kennedy. Full methods of this analysis including acyclic diagrams to represthe ent hypothesis are included.

## Results

Two thousand five hundred fourteen subjects in NOAR were identified to have genotype and mortality data available. Of these, 643 (25.6%) died during the study and 343 (53.3%) of these deaths were attributed to CV causes. Cohort characteristics are summarised in Table 1.

**Table 1:** Cohort characteristics

Characteristic	*N*/total (%)
Female sex, no. (%)	1650/2514 (65.6)
Age, median (IQR)	54 (43, 66)
Anti-CCP status; ever tested positive, No./total (%)	709/2196 (32.3)
Taking medications for diabetes, No. (%)	124/2514 (4.9)
Taking medications for hypertension, No, (%)	279/2514 (11.1)
Taking statin therapy	71/2514 (2.8)
Obese	559/2514 (22.2)
Current smoker	465/1783 (26.1)

HLA-DRB1 amino acids, haplotypes, or haplotype groups associated with RA susceptibility are also associated with CV mortality and this association is independent of sex, hypertension and obesity (Table 2). HLA-DRB1 polymorphisms encoding amino-acid haplotypes associated with an increased or decreased susceptibility to RA consistently show the same magnitude and direction of association for overall and cardiovascular mortality in IP and RA [6]. For example, the SEA-haplotype, associated with the lowest susceptibility to RA, and the best radiographic outcome, was found to be associated with decreased cardiovascular mortality (HR 0.67, 95% CI 0.47 to 0.94, *p*=0.023) [6]. The relative difference in mortality between carriers of the high susceptibility VKA haplotype and carriers of the SEA haplotype was significant (HR 1.67, 95% CI 1.13 to 2.48, *p*=0.01). The analysis was repeated adjusting for anti-CCP status and associations were found to be independent of this serological marker. The association with group 4 haplotypes remained statistically significant after adjustment for anti-CCP status (HR=0.74, 95% CI 0.60 to 0.92, *p*=0.007).

**Table 2:** Association statistics between genetic polymorphisms located within the HLA-DRB1 gene and disease mortality

Amino acid/haplotype/group	Inflammatory polyarthritis (IP)						Rheumatoid arthritis (RA)					
	All-cause mortality			Cardiovascular mortality			All-cause mortality			Cardiovascular mortality		
	Hazard ratio (95% CI)	***p*** value	***n***	Hazard ratio (95% CI)	***p*** value	***n***	Hazard ratio (95% CI)	***p*** value	***n***	Hazard ratio (95% CI)	***p*** value	***n***
Valine 11	1.16 (1.03, 1.30)	0.015	643 (2514)	1.10 (0.93, 1.30)	0.255	343 (2514)	1.10 (0.95, 1.28)	0.217	367 (1160)	0.96 (0.77, 1.19)	0.699	189 (1160)
Serine 11	0.83 (0.75, 0.94)	0.003	643 (2514)	0.82 (0.70, 0.96)	0.016	343 (2514)	0.83 (0.71, 0.97)	0.022	367 (1160)	0.86 (0.69, 1.07)	0.177	189 (1160)
Difference	1.26 (1.09, 1.45)	0.001		1.23 (1.01, 1.49)	0.038		1.22 (1.01, 1.47)	0.035		1.08 (0.83, 1.41)	0.559	
VKA haplotype	1.15 (0.99, 1.34)	0.073	579 (2328)	1.16 (0.94, 1.43)	0.158	310 (2328)	1.14 (0.94, 1.38)	0.190	333 (1078)	1.05 (0.79, 1.40)	0.715	167 (1078)
SEA haplotype	0.76 (0.59, 0.96)	0.024	579 (2328)	0.67 (0.47, 0.94)	0.023	310 (2328)	0.63 (0.44, 0.89)	0.009	333 (1078)	0.49 (0.28, 0.85)	0.011	167 (1078)
Difference	1.46 (1.11, 1.93)	0.007		1.67 (1.13, 2.48)	0.010		1.75 (1.19, 2.58)	0.005		2.09 (1.14, 3.84)	0.018	
Group 1	1.11 (0.98, 1.26)	0.101	579 (2328)	1.10 (0.93, 1.31)	0.266	319 (2328)	1.04 (0.89, 1.23)	0.622	333 (1078)	0.96 (0.76, 1.21)	0.720	167 (1078)
Group 4	0.78 (0.67, 0.90)	0.001	579 (2328)	0.73 (0.60, 0.89)	0.002	319 (2328)	0.76 (0.63, 0.93)	0.007	333 (1078)	0.72 (0.55, 0.96)	0.025	167 (1078)
Difference	1.31 (1.11, 1.54)	0.001		1.37 (1.09, 1.72)	0.007		1.27 (1.02, 1.58)	0.034		1.25 (0.91, 1.73)	0.168	

“Group 1” and “Group 4” refer to groups of haplotypes as previously defined in a previous publication [9]. Valine at position 11, the VKA haplotype and “group 1” haplotypes have previously been shown to be associated with the highest risk of susceptibility to RA [5]. Conversely, serine at position 11, the SEA haplotype and “group 4” haplotypes have been shown to be associated with the lowest risk [9]. Results are displayed as hazard ratios (HR) with 95% confidence intervals. The total number (*n*) of deaths is also displayed alongside the total number (*n*) of patients included in each analysis (in brackets). All models have been adjusted for cardiovascular risk factors namely; gender, hypertension and obesity. HR was not adjusted for other amino acids/haplotypes/groups. “Difference”: the difference in HR was calculated by the linear combination of the two HR obtained from a bivariate analysis (both amino acids/haplotypes/groups included in the same model). This represents the risk of death for the carriage of the highest risk susceptibility amino acid/haplotype/group, compared to the lowest risk amino acid/haplotype/group

**Fig. 1:**
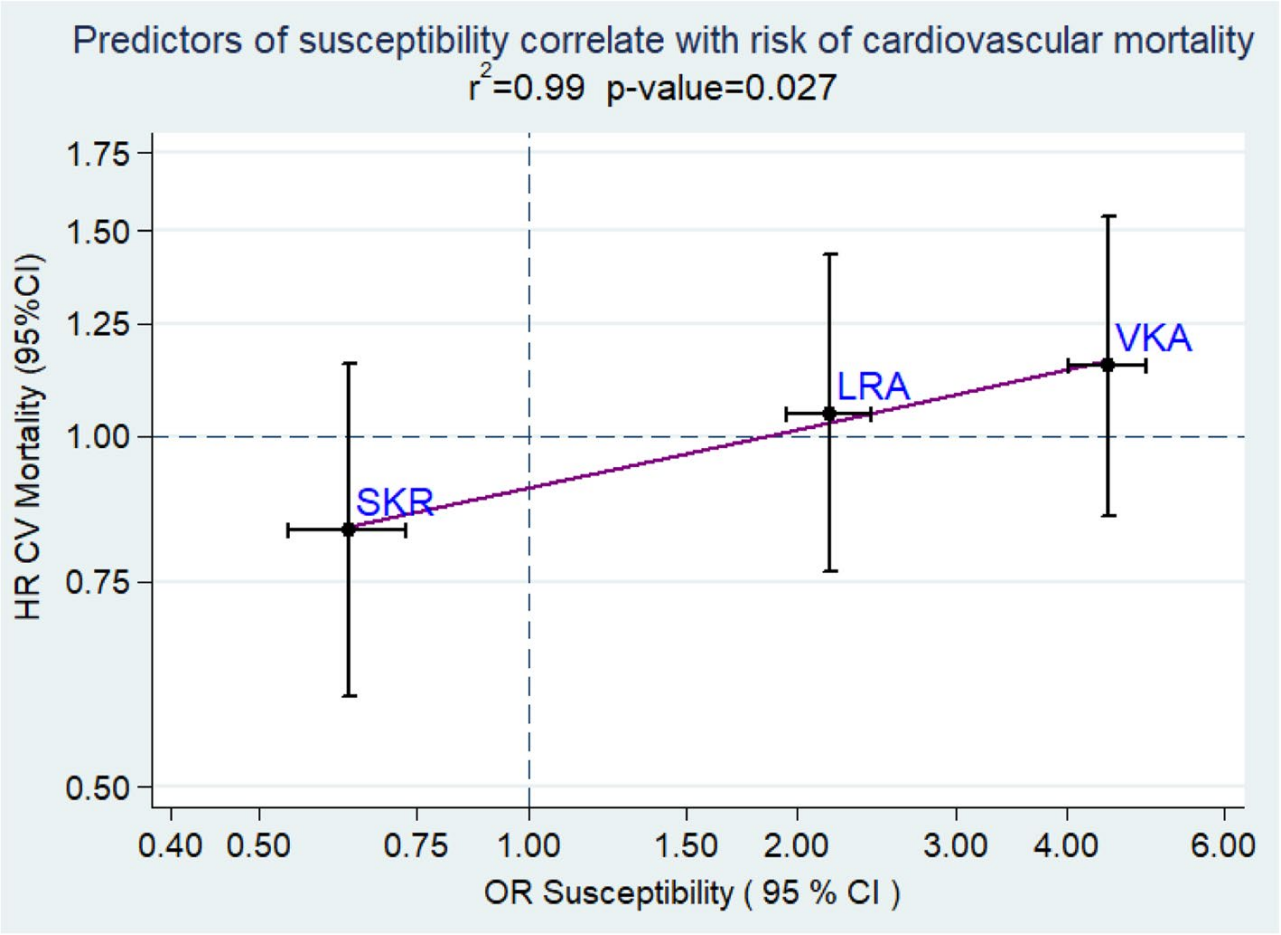
The effect sizes for susceptibility to rheumatoid arthritis correlate with the effect sizes for cardiovascular mortality in inflammatory polyarthritis in NOAR. This graph depicts the most frequent haplotypes occurring in the NOAR cohort, as defined by an allele frequency of over 12%. The *x*-axis shows the susceptibility to ACPA-positive RA expressed as odds ratios (see Raychaudhuri et al. [8]). The *Y*-axis shows cardiovascular mortality risk in inflammatory polyarthritis expressed as hazard ratios, which were derived from multi-variate cox-proportional hazard models adjusted for available cardiovascular risk factors: obesity, gender and presence of hypertension. Values are plotted on a logarithmic scale. A one-tailed *p* value was calculated using a linear regression model to determine the association between effect sizes (*β* coefficients) of susceptibility and cardiovascular mortality

HLA-DRB1 haplotypes can be ranked according to the magnitude of their association with RA susceptibility, and we have previously shown that this hierarchy is conserved for various measures of disease outcome and overall mortality [5, 8]. Our results show that this risk hierarchy is also conserved for CV mortality: HLA-DRB1 haplotypes that predispose to RA also predispose to increased CV mortality, independent of known CV risk factors (Fig. 1).

**Fig. 2:**
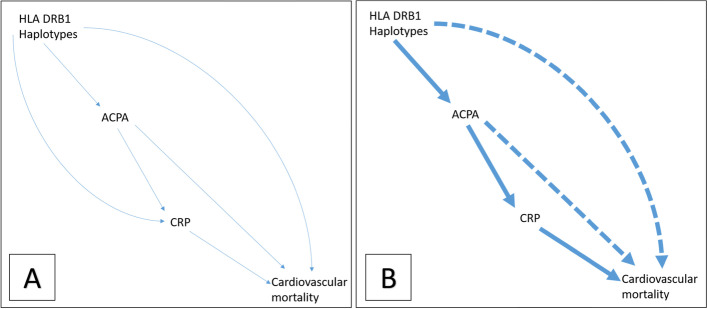
Mediation analysis. This figure shows acyclic graphs which depict results of mediation analysis in Table 3. **A** depicts hypothetical pathways and **B** shows pathways identified as significant from the analysis. The main proposed pathways are highlighted in bold. Alongside Table 3, it suggests some of the association between genetic risk (HLA DRB1 haplotypes) and cardiovascular mortality in inflammatory polyarthritis is independent of anti-CCP and CRP

We next performed mediation analysis and the results of tstep-by-stepstep analysis are shown in Table 3. Genetic markers (serine at position 11; Ser^11^) are shown to be associated with CRP (−2.49 (−4.13, −0.85), *p*=0.003) and in a separate model, with anti-CCP (−0.85, (−1.00, −0.70), *p*=0.000). However, in a model containing both of these factors, Ser^11^ is no longer associated with CRP (*p*=0.614), suggesting the association between Ser^11^ and CRP is fully mediated by anti-CCP status. We found that Ser^11^ is associated with cardiovascular mortality, and some of this association is independent of anti-CCP and CRP. This is demonstrated in a final model, containing Ser^11^, CRP and anti-CCP, where Ser^11^ remained protective (0.83 [ 0.69–1.00], *p*=0.048). This suggests there is a non-systemic pathway through which genetic markers may exert an effect on CV mortality. These findings are depicted in cyclic diagrams in Fig. 2.

**Table 3:** Mediation analysis

***Step 1: Association of serine at position 11 and mediators***				
Model	Variable	B coefficient	*p* value	Interpretation
Linear regression: serine 11, CRP	CRP	−2.49 (−4.13, −0.85)	0.003	Suggests serine 11 is associated with CRP
Logistic regression: serine 11, anti-CCP	Anti-CCP	−0.85 (−1.00, −0.70)	0.000	Suggests serine 11 is associated with anti-CCP status
Multivariate regression: serine 11, anti-CCP and CRP	CRP	0.00 (0.00, 0.00)	0.614	Suggests association between serine 11 and CRP fully mediated by ACPA status.
	Anti-CCP	−0.37 (−0.43, −0.30)	0.000	
***Step 2: Association of serine at position 11 and cardiovascular mortality***				
Model	Variable	Hazard ratio	*p* value	Interpretation
Model predicting CV mortality (controlled for cv risk factors) with serine 11	Serine 11	0.82 (0.70, 0.96)	0.016	Suggests association between serine 11 and CV mortality
***Step 3: Association of Serine at position 11 and cardiovascular mortality***				
Model	Variable	Hazard ratio	*p* value	Interpretation
Model predicting CV mortality (controlled for cv risk factors) with serine 11, CRP	CRP	1.01 (1.00, 1.01)	0.001	Suggests association of CRP and CV mortality
	Serine 11	0.83 (0.70, 0.99)	0.034	Suggests association of serine 11 and CV mortality, independent of CRP
Model predicting CV mortality (controlled for cv risk factors) with serine 11, ACPA	Anti-CCP	1.50 (1.18, 1.92)	0.001	Suggests association of anti-CCP and CV mortality
	Serine 11	0.81 (0.68, 0.98)	0.027	Suggests association of serine 11 and CV mortality, independent of anti-CCP
Model predicting CV mortality (controlled for cv risk factors) with serine 11, ACPA, CRP	CRP	1.00 (1.00, 1.01)	0.005	Suggests association of CRP and CV mortality, independent of anti-CCP
	Anti-CCP	1.40 (1.08, 1.81)	0.011	Suggests association of anti-CCP and CV mortality
	Serine 11	0.83 (0.69, 1.00)	0.048	**Suggests association of serine 11 and CV mortality, independent of anti-CCP and CRP**
***Other relevant models***				
Model	Variable	*B* coefficient	*p* value	
Regression CRP, anti-CCP	Anti-CCP	12.71 (10.55, 14.87)	0.000	
Association of serine at position 11 and rheumatoid factor was also tested which showed a significant association. However, when adjusted for anti-CCP, this association no longer stood. For this reason, the rheumatoid factor was not included in further mediation analysis. See below:				
Model	Variable	*B* coefficient	*p* value	
Regression model serine 11, rheumatoid factor and anti-CCP	Anti-CCP	− 0.35 (− 0.42, − 0.28)	0.000	
	Rheumatoid factor	− 0.01 (− 0.09, 0.06)	0.691	

The results of mediation analysis which was performed as per principles according to Baron and Kennedy. This was performed in steps as shown in order to determine whether the association of the above genetic factors with CV mortality was likely to be through intermediate parameters of inflammation. Proposed pathways are summarised in Fig. 2

## Discussion

It has been shown that CV mortality in RA has, in part, a genetic basis. “The shared epitope” (SE) has been previously shown to be associated with CV mortality, and this association is independent of autoantibody status [6, 7, 17]. To our knowledge, the association between amino acids at positions 11, 71 and 74 of HLA-DRB1 and CV mortality in RA has not previously been explored. We demonstrate that these originally reported genetic associations between HLA-DRB1 polymorphisms and disease susceptibility and severity are also associated with CV mortality.

Valine at position 11 of HLA-DRB1, outside of the SE, was shown to have the highest association with disease susceptibility, radiological damage and all-cause mortality, whilst serine at this position was most protective [8–12]. In this study, we demonstrate that the relative differences in CV mortality between carriers of high and low susceptibility genetic risk factors are similar to that of all-cause mortality.

There are some possible explanations behind the associations between HLA-DRB1 haplotypes and CV mortality in inflammatory polyarthritis patients. HLA-DRB1 haplotypes increase susceptibility to RA which in turn is well known to increase CV morbidity and mortality. However, when restricting the analysis to those who satisfied criteria for RA only, effect sizes for highest risk haplotype (VKA) and lowest risk (SEA) increase. This suggests that there is a mechanism through which HLA independently influences the risk of CV death in these patients, as opposed to solely through the development of RA.

One possible explanation of the influence of HLA-DRB1 haplotypes on this risk is through increased systemic inflammation [18]. In mediation analysis, the effect of serine at position 11 on CV mortality was partially but not completely mediated through anti-CCP status and CRP. This suggested an independent non-systemic pathway through which HLA-DRB1 haplotypes are associated with CV mortality, which is yet to be elucidated.

Another possible mechanism behind the association of HLA-DRB1 haplotypes with CV mortality may be as a result of increased disease severity rather than increased susceptibility. Those with severe disease are more likely to carry risk haplotypes as previously demonstrated [12]. If those patients are also more predisposed to CV mortality, this could also explain the results found.

The exact mechanism of action underlying the association of HLA-DRB1 haplotypes with susceptibility to and outcome of RA remains a matter of debate. However, mounting evidence suggests that haplotypes associated with high susceptibility and more severe outcomes carry amino acids within their peptide-binding groove which will increase affinity to autoantigenic peptides [19]. The presentation of autoantigenic peptides to CD4+ T and the activation of these cells are likely to play a crucial role in the onset and maintenance of the disease [20].

To our knowledge, there are no other studies looking specifically at CV mortality and HLA-DRB1 haplotypes defined by positions 11, 71 and 74. However, there is a study involving a male-predominant cohort of US veterans, which explored the association with these haplotypes and all-cause mortality [21]. Although they did not find an association with VKA and SEA haplotypes and all-cause mortality, it is possible the study was underpowered (1443 participants versus 2514 in NOAR). In addition, the NOAR participants were more likely to be female, had a lower comorbidity burden and lower smoking prevalence and were much less likely to be on disease-modifying therapy at baseline. These factors across the two populations could also explain the differences in results for overall mortality.

A particular strength of this study is that NOAR is a large inception cohort of patients with inflammatory polyarthritis with the availability of genetic and mortality data. There are some limitations to this study: information on CV mortality was derived from death certification, which may be inaccurate. The data on covariates was collected at baseline, and it is possible that the status of these may change through the course of follow-up. However, the aim of this study was to informthe prediction of those at highest risk at disease onset, making this a greater representation of a clinical setting. Clearly, replication of these findings in independent cohorts would be required to confirm these findings and to determine the role of patients’ HLA typing in the assessment of CV risk in RA.

Treatment of RA has changed considerably since the conception of NOAR, and there is evidence that effective treatment of disease activity reduces the risk of premature cardiovascular death. Further investigation would be required to evaluate whether this also modulates the association between HLA-DRB1 haplotypes and CV mortality.

## Conclusions

CV disease and mortality remain a significant challenge in the management of RA. Understanding the mechanisms underpinning genetic associations of mortality in RA could help enable risk stratification of patients from disease presentation. Our findings show HLA-DRB1 haplotypes associated with susceptibility to RA also predispose to increased risk of CV mortality in IP, independent of known CV risk factors. Notably, associations were persistent after adjustment of anti-CCP status. This suggests in the future that genetic factors will add to the prediction of the risk of cardiovascular mortality beyond serological markers. The clinical utility of whether HLA typing in informing management of cardiovascular risk remains to be explored, but would likely form part of a larger tool including clinical and serological predictors.

## Data Availability

The data that support the findings of this study are available from the corresponding author, [SV], upon request, wherever legally and ethically possible.

## Abbreviations

RA: Rheumatoid arthritis
IP: Inflammatory polyarthritis
CVD: Cardiovascular disease
CV: Cardiovascular
CRP: C-reactive protein
SE: Shared epitope
CI: Confidence interval
CCP: Anti-cyclic citrullinated peptide
HLA: Human leukocyte antigen
NOAR: Norfolk Arthritis Register
Ser11: Serine at position 11
